# Matrix stiffness in osteoarthritis: from mechanism introduction to biomaterial-based therapies

**DOI:** 10.3389/fendo.2025.1571502

**Published:** 2025-05-08

**Authors:** Kai Huang, Haili Cai

**Affiliations:** ^1^ Department of Orthopaedics, Tongde Hospital of Zhejiang Province, Hangzhou, China; ^2^ Department of Ultrasound Medicine, The 903rd Hospital of The People's Liberation Army, Hangzhou, Jiangsu, China

**Keywords:** osteoarthritis, cartilage, matrix stiffness, signaling pathways, chondrocytes

## Abstract

Osteoarthritis (OA), the most prevalent joint disorder associated with aging, is characterized by impaired extracellular matrix (ECM) synthesis and the degradation of articular cartilage. It is influenced by various factors, including aging and mechanical stress (such as traumatic injury). Increasing evidence suggests that alterations in cartilage stiffness occur during OA progression, particularly at its onset. This review comprehensively examines how aging and mechanical stress contribute to ECM stiffening, a precursor to irreversible cartilage degradation. We also discuss how increased matrix stiffness disrupts the homeostatic balance between chondrocyte catabolism and anabolism and the mechanotransduction pathways involved in cartilage stiffening. Furthermore, the potential of cartilage engineering to target the stiffness of synthetic materials is explored as a promising approach to advancing cartilage repair and regeneration in OA. A deeper understanding of this research area may not only lead to more innovative strategies for early OA detection and diagnosis but also offer novel insights into OA treatment and prognosis.

## Introduction

Osteoarthritis (OA) is a complex degenerative condition influenced by factors such as aging, obesity, and trauma. OA begins at the molecular level and progressively disrupts the structure and function of articular cartilage, ultimately leading to irreversible damage (1). While some medications can alleviate inflammation and pain associated with OA, few have shown efficacy in halting or reversing the disease’s progression. This challenge is further exacerbated by the limited regenerative capacity of cartilage, leading to a shortage of clinically effective interventions for OA.

Matrix stiffness significantly influences cell proliferation, phenotype, and differentiation among the physical changes observed in OA (2). Research shows that matrix stiffness directly affects chondrocyte behavior and phenotype, particularly in laboratory conditions (3). The stiffening of the extracellular matrix (ECM) is a critical factor in cartilage aging and a major contributor to the development of knee OA (4). However, the precise molecular and cellular mechanisms underlying age-related changes in biomechanical properties remain largely unexplored. Furthermore, there is a notable gap in research focused on strategies targeting the downstream cellular response to cartilage matrix stiffness in OA treatment. This ongoing lack of effective therapeutic targets continues to challenge clinicians, often resulting in further cartilage degeneration or the need for total joint replacement (5, 6).

## The upstream molecular mechanisms triggering cartilage matrix stiffening

Understanding the upstream molecular mechanisms that lead to cartilage matrix stiffening is essential for grasping the progression of OA. Alterations in ECM composition and chondrocyte behavior, driven by aging and mechanical stress, are key factors. These changes disrupt the balance between catabolic and anabolic processes in cartilage, leading to increased stiffness, a precursor to cartilage degradation and symptomatic OA.

### Ageing-related cartilage matrix stiffening

#### Changes in ECM proteins

As articular cartilage ages, it undergoes substantial structural, compositional, and mechanical changes that lead to increased extracellular matrix (ECM) stiffness (7). In both mice and humans, this age-related stiffness is observed to be two to three times greater than that in younger adults (8). Changes in ECM proteins, such as collagen and proteoglycans, drive nonenzymatic collagen cross-linking and shorten aggrecan molecules (4, 9). Excessive collagen cross-linking increases cartilage stiffness, making it more brittle and susceptible to fatigue failure (10). The degradation of aggrecan further exacerbates this process by reducing sugar side chains and the ability to bind water (11).

#### Advanced glycation end-products

Additionally, elevated levels of advanced glycation end-products (AGEs) have been associated with decreased anabolic activity, which promotes further nonenzymatic collagen cross-linking, thereby increasing ECM stiffness (12). AGEs and their receptors in articular tissue provide valuable insights into the biochemical and biomechanical changes that contribute to OA pathogenesis (13). The accumulation of AGEs is linked to increased cellular senescence, which reduces chondrocyte metabolic and biosynthetic functions, promoting cartilage degeneration and OA progression (13). Studies have shown that the upregulation of AGEs in aging joints accelerates matrix stiffening through collagen cross-linking and the loss of glycosaminoglycans (GAGs) (4). *In vitro* experiments with threose have demonstrated increased AGE cross-linking in cartilage, leading to enhanced collagen network stiffness and contributing to age-related collagen degradation and increased susceptibility to damage (10).

### Trauma-related cartilage matrix stiffening

#### Mechanical stress

Mechanical stress resulting from joint instability and injury is another significant risk factor for the development of osteoarthritis (OA). Traumatic events can accelerate the onset of OA, suggesting that matrix cross-linking in post-traumatic OA (PTOA) can occur independently of the advanced glycation end-products (AGEs) accumulation typically seen in age-related OA. While spontaneous OA is more common in older individuals, PTOA often affects younger adults, leading to irreversible cartilage degradation due to injury. PTOA may arise from direct damage to cartilage or trauma to the joint.

#### Lysyl oxidase secretion

Mechanical stress triggers the extracellular secretion of lysyl oxidase (LOX), an enzyme that significantly increases collagen matrix stiffness (Young’s modulus). Kim et al. identified mechanical stress as a key factor in cartilage matrix stiffening in OA (4). Their *in vitro* studies showed that LOX secretion enhances collagen matrix stiffness, with LOX-treated chondrocytes exhibiting OA-associated gene expression (4). *In vivo*, joint instability from medial meniscus destabilization (DMM) elevated LOX levels, which preceded MMP-13 expression and cartilage degradation. These findings suggest that matrix stiffening is an early event in trauma-related OA progression. In contrast, Chery et al. observed a moderate reduction in the modulus of DMM-treated murine cartilage, likely due to differences in study focus: Kim et al. measured the elastic modulus of LOX-CM gel used for culturing chondrocytes, while Chery et al. assessed cartilage tissue modulus three days post-DMM without considering ECM-degrading enzyme activity (14, 15). The immediate ECM degradation following DMM likely explains the reduced modulus observed. Despite these discrepancies, both studies highlight the critical role of ECM micromechanics in early PTOA, emphasizing the detrimental cycle of cartilage degeneration caused by impaired mechanosensitive responses in chondrocytes.

## The downstream molecular mechanisms of cartilage matrix stiffening

**Figure 1:**
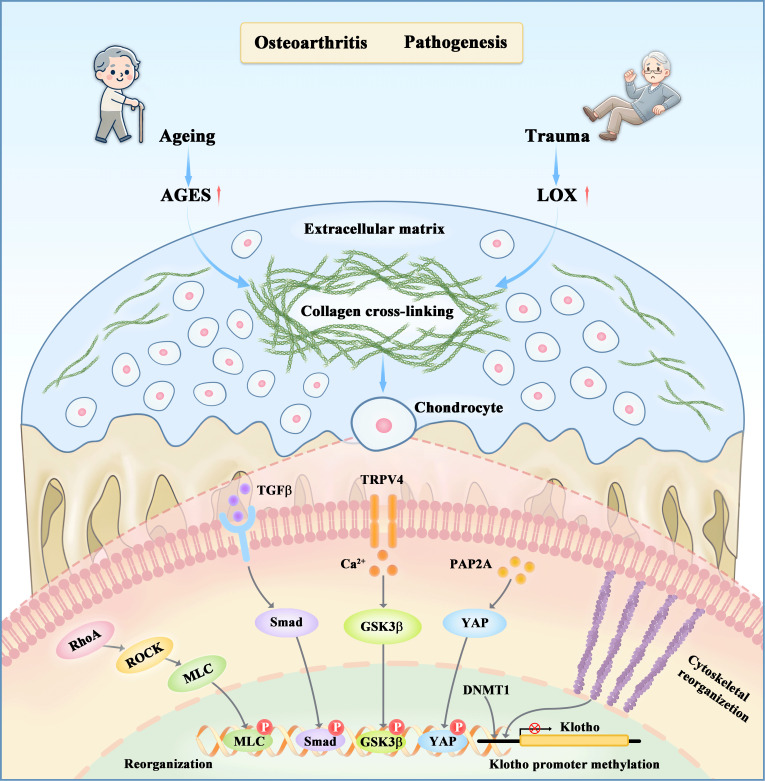
The regulatory effects of matrix stiffness on chondrocyte metabolism through both upstream and downstream mechanisms. Aging and mechanical stress are two primary factors that initiate osteoarthritis by inducing collagen crosslinking in cartilage tissues, resulting in extracellular matrix stiffening. This stiffening disrupts the catabolic-anabolic balance of chondrocyte metabolism through various mechanotransductive pathways, such as RhoA/ROCK, PAP2A/YAP, TGFβ/Smad, and Ca2+ channels TRPV4, as well as epigenetic modifications. Subsequent events include the phosphorylation of YAP, MLC, Smad3, and GSK3β, which activate genes involved in cytoskeleton reorganization, inflammation, and chondrocyte metabolism.

The downstream molecular and cellular mechanisms that contribute to cartilage matrix stiffening, an early event in both spontaneous OA and PTOA, are not yet fully understood. Research shows that stiff substrates enhance chondrocyte stress fiber formation and alter cellular morphology, highlighting the importance of mechanotransductive signaling in regulating cellular phenotypes in response to matrix stiffness (14, 16). Several key mechanotransduction pathways have been identified in the initiation of OA (**Figure 1**).

### RhoA/ROCK signaling

The Ras homolog gene family member A (RhoA) and its downstream effector, Rho-associated protein kinase (ROCK), play crucial roles in various cellular processes, particularly in the pathogenesis of osteoarthritis (OA). Aberrant activation of the RhoA/ROCK pathway is strongly associated with OA development. This pathway is vital for regulating stress fiber formation and cytoskeletal reorganization within chondrocytes, both of which are key contributors to OA progression (17).

Recent studies have highlighted the differential behavior of chondrocytes in two-dimensional (2D) versus three-dimensional (3D) environments. In 2D cultures, increased matrix stiffness leads to abnormal RhoA/ROCK signaling, resulting in enhanced stress fiber formation and elevated catabolic activity. Kim et al. demonstrated that increased matrix stiffness activates the RhoA/ROCK pathway, leading to increased chondrocyte catabolism and decreased anabolism. Activation of the RhoA-ROCK-MLC axis drives these molecular changes, and inhibiting this axis can effectively prevent the stiffness-induced imbalance between catabolic and anabolic activities (4). Appleton et al. further found that stress fiber formation and contractility, driven by RhoA/ROCK signaling, negatively impact chondrocyte phenotype by altering Sox9 expression and disrupting actin organization. They proposed that targeting the RhoA/ROCK pathway could mitigate cartilage degeneration, promote cartilage maintenance, and support repair processes (18). Additionally, Liang et al. (19) revealed that leptin activates the RhoA/ROCK pathway, leading to significant cytoskeletal reorganization in chondrocytes. This reorganization alters cell morphology, causing changes such as flattening, elongation, and hypertrophy, and promotes the formation of stress fibers. These morphological and functional changes can disrupt normal cellular activities, shifting chondrocyte metabolism from anabolic (building up) to catabolic (breaking down) processes. However, in 3D environments, the effects of stiffness on RhoA/ROCK signaling can be attenuated or even reversed, with chondrocytes exhibiting less catabolic behavior in stiffer matrices. This suggests that the dimensionality of the culture system significantly influences the cellular response to mechanical cues, which is crucial for interpreting *in vitro* findings in the context of *in vivo* tissue environments.

### TGFβ/SMAD3 signaling

Transforming growth factor-beta (TGFβ) is a versatile cytokine that plays a critical role in regulating cell proliferation, differentiation, and extracellular matrix production. Upon binding to its receptors on the cell surface, TGFβ initiates a signaling cascade that results in the phosphorylation and activation of SMAD3. Once activated, SMAD3 translocates to the nucleus, where it regulates gene expression related to chondrocyte metabolism. Research has shown that matrix stiffness can modulate the TGFβ/SMAD3 signaling pathway, significantly influencing chondrocyte metabolic responses (20, 21). For example, recent studies have demonstrated that chondrocytes cultured on substrates with a specific stiffness (0.5 MPa) exhibit enhanced differentiation. This is evidenced by increased deposition of proteoglycans and elevated expression levels of chondrogenic markers such as Sox9, Col2α1, and aggrecan. This process is mediated by the stiffness-sensitive induction of TGFβ1, which promotes SMAD3 phosphorylation, its nuclear localization, and subsequent transcriptional activity. These findings highlight the synergistic interaction between extracellular matrix (ECM) stiffness and TGFβ, revealing how chondrocytes integrate both physical and biochemical signals to coordinate their responses within the microenvironment (22).

### YAP signaling

Yes-associated protein (YAP) is a well-characterized mechanotransducer that plays a crucial role in transmitting mechanical signals from surrounding cells to the nucleus (23). In articular cartilage, a load-bearing tissue, mechanical forces such as pressure, tensile stress, and fluid shear stress are mediated through the Hippo/YAP pathway (24, 25). Zhang et al. discovered that in stiffer microenvironments, YAP mediates chondrocyte catabolism by promoting YAP dephosphorylation and its translocation to the nucleus. Both *in vivo* and *in vitro* experiments have shown that increased nuclear YAP levels are associated with reduced cartilage matrix deposition in osteoarthritis (OA), particularly in cases where the microenvironment is stiffer (26). Further studies have demonstrated that genetic deletion of YAP or pharmacological inhibition of its nuclear translocation can enhance chondrocyte anabolism in stiff scaffolds, leading to reduced cartilage degradation in OA animal models. Similarly, Zhong et al. identified a clear correlation between substrate stiffness and changes in chondrocyte phenotype, with YAP localization shifting in response to these changes. Specifically, nuclear YAP levels were significantly lower on softer substrates, which coincided with an increase in Col2α1 expression compared to stiffer substrates (27). Recent evidence highlights the crosstalk between the TGFβ/SMAD3 and YAP signaling pathways in osteoarthritis (OA). Runx1 has been identified as a key regulator that coordinates multiple signaling pathways, including TGFβ/Smad, Hippo/YAP, and Wnt/β-catenin (28). It integrates these pathways to regulate articular cartilage homeostasis and contribute to cartilage degradation in OA (29). Specifically, Runx1 influences the expression of YAP and p-Smad2/3, which are critical mediators in these signaling pathways. Loss of Runx1 in cartilage leads to a marked reduction in YAP protein levels and p-Smad2/3 expression. This suggests that Runx1 is essential for maintaining the activity of both the TGFβ and Hippo/YAP pathways. In the absence of Runx1, the function of these pathways is impaired, resulting in cartilage degradation and osteophyte formation in OA. Notably, the TGFβ pathway plays a pivotal role in maintaining cartilage integrity by regulating the phosphorylation of Smad2/3, a key step in its activation (30).

### α-Klotho signaling

A recent systematic review has highlighted that elevated inflammation, increased senescence, and impaired autophagy are common factors associated with age-induced knee osteoarthritis (OA) in mice (31), all of which are downstream of α-Klotho (32, 33). Moreover, studies have demonstrated that α-Klotho overexpression can delay cartilage degeneration in a post-traumatic OA (PTOA) model. Earlier *in vitro* and *in vivo* investigations have suggested that α-Klotho overexpression can alleviate chondrocyte dysfunction and cartilage degeneration in a PTOA model (34). Additionally, a separate study has shown that combining α-Klotho overexpression with soluble TGF-β supplementation can enhance chondrocyte health and cartilage integrity in a chemically-induced OA model (35). Iijima et al. (36) found that young chondrocytes cultured on rigid substrates exhibited aging characteristics compared to those cultured on flexible substrates, as evidenced by decreased levels of type II collagen and aggrecan. This change coincided with a reduction in α-Klotho protein levels. In contrast, aged chondrocytes cultured on flexible substrates showed elevated levels of type II collagen and α-Klotho compared to those cultured on rigid substrates.

### Calcium signaling

Recent studies have examined the influence of extracellular microenvironment stiffness on intracellular Ca^2+^ signaling in chondrocytes and its implications for osteoarthritis (OA) progression. The TRPV4 ion channel, which facilitates the passage of calcium ions, plays a pivotal role in regulating mechanical stress in cartilage. Alterations in TRPV4 expression have been found to affect OA progression differently depending on age and traumatic injury (37). Additionally, ECM viscoelasticity has been shown to modulate TRPV4 activity in healthy chondrocytes, leading to a decrease in intracellular Ca^2+^ in fast-relaxation matrices and an increase in intracellular Ca^2+^ in low-relaxation matrices (38). Similarly, Zhang et al. (3) demonstrated that different substrate stiffness levels induce distinct dynamic deformations in chondrocytes during cell swelling and recovery processes. Specifically, chondrocytes exhibit accelerated swelling on soft substrates but slower recovery compared to stiff substrates when subjected to hypo-osmotic challenges. Their research also revealed that stiff substrates intensify cytosolic Ca^2+^ oscillations in chondrocytes under iso-osmotic conditions. Soft substrates notably influence Ca^2+^ oscillations during cell swelling, while stiff substrates enhance cytosolic Ca^2+^ oscillations during cell recovery. Moreover, the TRPV4 channel contributes to chondrocyte perception of substrate stiffness by modulating Ca^2+^ signaling in a manner dependent on substrate stiffness.

## Clinically relevant evidence on cartilage ECM stiffening

Although no clinical trials have yet been conducted on the role of cartilage matrix stiffness in the pathogenesis of OA, experimental findings based on human samples have already demonstrated that cartilage matrix stiffness can accelerate chondrocyte senescence and promote cartilage degradation. Fu et al. (39) demonstrated that human OA cartilage exhibits increased matrix stiffness compared to normal cartilage. This stiffening was positively correlated with the degree of cartilage damage, supporting the hypothesis that matrix stiffness is a critical factor in OA progression. In human samples, senescent chondrocytes exhibited higher expression of markers such as p16INK4a, p21, and p53. These findings suggest a strong association between ECM stiffness and the senescence phenotype in chondrocytes, further linking matrix stiffness to cartilage degeneration in OA. Liu et al. (40) found valuable insights into the mechanistic relationship between matrix stiffness, chondrocyte behavior, and the progression of intervertebral disc degeneration in humans. Human cartilage endplate (CEP) samples from 48 patients undergoing spinal fusion surgery were analyzed. The matrix stiffness of the CEPs increased with the degeneration grade, from mild (Grade 2) to severe (Grade 6), and was positively correlated with the degree of IDD. As the degeneration progressed, the elastic modulus of the matrix increased, indicating stiffer matrices in more degenerated CEPs. This change in matrix stiffness was linked to collagen disarrangement and the degeneration of cartilage in these human samples.

## Matrix stiffness as a promising microenvironmental factor in cartilage engineering

Matrix-assisted autologous chondrocyte implantation (MACI) has garnered considerable attention for its potential to treat articular cartilage lesions in osteoarthritis (OA), as well as its ability to improve biomimetic substrate designs that promote extracellular matrix (ECM) deposition by transplanted chondrocytes (41). By cultivating chondrocytes *in vitro* within scaffolds of varying stiffness, researchers have developed a reliable method to study the impact of matrix stiffness on chondrocyte metabolism. Various studies have explored the role of scaffold stiffness in regulating cytoskeletal organization, metabolic activity, and chondrocyte proliferation on these substrates (42–44). Shojarazavi et al. (45) developed an injectable hydrogel based on alginate and cartilage ECM, enhanced with silk fibroin nanofibers (SFN), to improve cartilage tissue engineering. By optimizing SFN concentrations, they achieved a targeted mechanical stiffness of 102.97 ± 4.75 kPa. Cell viability assessments and measurements of glycosaminoglycan (GAG) and collagen II content revealed that this hydrogel effectively mimicked the natural cartilage environment, highlighting its significant potential. The morphology of chondrocytes is intricately controlled by the formation and organization of their cytoskeletal fibers (46). Chondrocytes are sensitive to the stiffness of their surrounding scaffolds, which induces structural adjustments in their cytoskeletons to align with the mechanical properties of their microenvironment (47). Studies using atomic force microscopy (AFM) to evaluate the mechanical properties of scaffolds have shown that stiffer matrices lead to increased elastic modulus and other viscoelastic parameters of chondrocytes. Furthermore, research has indicated that chondrocytes cultured on hydrogels with higher Young’s modulus exhibit a rise in cellular stiffness (48). These insights are valuable for optimizing the design of scaffolds for cartilage repair, which could further enhance outcomes for OA regeneration.

## Prolotherapy for ECM and cartilage regeneration

Prolotherapy, particularly dextrose prolotherapy, has shown potential in promoting tissue regeneration by stimulating local inflammatory responses and collagen deposition (49). Studies have suggested that dextrose injections can stimulate the production of growth factors, such as platelet-derived growth factor, transforming growth factor-β, and basic fibroblast growth factor, which help repair damaged cartilage and surrounding tissues (50, 51). While the precise mechanisms of prolotherapy remain debated, its potential in ECM and cartilage regeneration is promising, with multiple studies highlighting its efficacy in pain reduction and improved function for OA patients (52, 53). Further research and inclusion of such treatments in clinical practice could enhance current therapies for cartilage regeneration.

## Conclusions

Osteoarthritis (OA) is a prevalent knee joint disorder worldwide, with age, obesity, and trauma recognized as primary risk factors. While medications exist to reduce inflammation and manage OA-associated pain, few have demonstrated efficacy in halting or improving disease progression. The limited success of non-surgical OA treatments can be attributed, at least in part, to gaps in our understanding of the translational relevance of preclinical OA models and an incomplete understanding of the molecular mechanisms underlying disease pathogenesis. This review analyzes recent developments in understanding how changes in cartilage stiffness contribute to OA progression. Aging and mechanical strain, which are associated with joint instability and trauma—primary risk factors for initiating OA—have been identified as sources of matrix collagen crosslinking, leading to cartilage stiffness. The relevant mechanotransductive signaling pathways following cartilage stiffening are discussed, along with their involvement in chondrocyte anabolism and catabolism. Although cartilage ECM stiffening is an early molecular event in OA, current methods for detecting the degree of cartilage matrix sclerosis are limited to laboratory techniques, such as nano-AFM microscopy. There is currently a lack of effective clinical detection tools, which further hinders its clinical translation. It is hoped that increased attention and understanding in this research area will lead to the development of more innovative methods for early OA detection and diagnosis, as well as promising treatment and prognosis strategies.
